# CCR4-dependent reduction in the number and suppressor function of CD4^+^Foxp3^+^ cells augments IFN-γ-mediated pulmonary inflammation and aggravates tuberculosis pathogenesis

**DOI:** 10.1038/s41419-018-1240-3

**Published:** 2018-12-21

**Authors:** Thais B. Bertolini, Annie R. Piñeros, Rafael Q. Prado, Ana Flávia Gembre, Leandra N. Z. Ramalho, José Carlos Alves-Filho, Vânia L. D. Bonato

**Affiliations:** 1 Department of Biochemistry and Immunology, Ribeirao Preto Medical School, University of Sao Paulo, Ribeirao Preto, Sao Paulo, Brazil; 2 Department of Pathology, Ribeirao Preto Medical School, University of Sao Paulo, Ribeirao Preto, Sao Paulo, Brazil; 3 Department of Pharmacology, Ribeirao Preto Medical School, University of Sao Paulo, Ribeirao Preto, Sao Paulo, Brazil

## Abstract

Chronic pulmonary inflammation marked predominantly by CD4^+^IFN-γ^+^ cells is the hallmark of tuberculosis pathogenesis in immunocompetent adults, who are substantially affected by this disease. Moreover, CD4^+^Foxp3^+^ cell-mediated suppression contributes to infection susceptibility. We addressed the role of CD4^+^Foxp3^+^ cells in tuberculosis pathogenesis, because this aspect has not been addressed during chronic infection. We targeted CCR4, which induces the influx of CD4^+^Foxp3^+^ cells into the lungs. CCR4^−/−^ mice exhibited a lower frequency of CD4^+^Foxp3^+^ cells at 15, 30, and 70 days of infection than their wild-type counterparts. However, only at 70 days of infection was an exacerbated IFN-γ-mediated immune response associated with apparent tuberculosis pathogenesis and susceptibility. In addition, CCR4^−/−^ mice exhibited a decrease in the suppressor function of CD4^+^Foxp3^+^ cells. Adoptive transfer of Foxp3^+^ cells into infected CCR4^−/−^ mice restored pulmonary inflammation and bacterial load to levels observed in wild-type mice. Our findings suggest that CD4^+^Foxp3^+^ cells play a time-dependent role in tuberculosis and highlight that CCR4 plays a critical role in the balance of IFN-γ-mediated inflammation by regulating the influx and function of CD4^+^Foxp3^+^ cells. Our findings are translationally relevant, as CD4^+^Foxp3^+^ cells or CCR4 could be a target for immunotherapy, considering the heterogeneity of tuberculosis in immunocompetent adults.

## Introduction

The treatment of tuberculosis remains a great challenge, and researchers are attempting to develop new vaccines that can confer stronger protection than the BCG vaccine and prevent the progression of active pulmonary disease in adults^1^. With the first observations of HIV (human immunodeficiency virus) infection in 1981, there was a remarkable increase in the number of individuals co-infected with HIV and *Mycobacterium tuberculosis*^2^. However, the Global Tuberculosis Report indicated that in 2016, there were 1.3 million tuberculosis deaths among HIV-negative individuals and 374,000 deaths among HIV-positive individuals^3^. Therefore, the majority of infected subjects who develop active tuberculosis worldwide are immunocompetent individuals^3^.

The hallmark of the protective immune response against tuberculosis is the differentiation of INF-γ-producing CD4^+^ cells and the activation of inflammatory M1 macrophages^4,5^. *M. tuberculosis* infection is a powerful stimulus for the differentiation of CD4^+^IFN-γ^+^ cells^5^. Although CD8^+^ T cells, NK (natural killer) cells, γδ T cells and CD1-restricted T cells also secrete IFN-γ after recognizing *M. tuberculosis* antigens, they do not compensate for the secretion of this cytokine in the absence of CD4^+^ cells^5,6^. IFN-γ stimulates the antimicrobial potential of macrophages, such as NO (nitric oxide) production^7^, induces phagosome-lysosome fusion^8,9^ and activates the autophagy pathway, which plays a protective role in mycobacterial infection^10^. The protective role of IFN-γ in tuberculosis has been demonstrated by clinical studies, and deficiency in the gene encoding IFN-γ increases susceptibility to mycobacterial infections^11^. In addition, mice deficient for the expression of IFN-γ succumb to *M. tuberculosis* infection^12,13^.

However, CD4^+^IFN-γ^+^ cells are also associated with tuberculosis pathogenesis in tuberculosis-associated immune reconstitution inflammatory syndrome, which is recurrent in a subset of individuals co-infected with HIV and *M. tuberculosis* treated with antiretroviral therapy as well as in immunocompetent adults^14–16^. Levels of IFN-γ in the bronchoalveolar lavage fluid of patients with active tuberculosis are correlated with disease severity^17^. Berry et al. described the increase in inducible IFN-γ gene expression in patients with active tuberculosis compared with healthy and latently infected subjects^18^. We reported that high levels of IFN-γ induced by immunization with *M. tuberculosis* culture filtrate proteins (CFP) plus CpG oligodeoxynucleotides are associated with extensive lung inflammation and do not confer protection against *M. tuberculosis* challenge compared with non-immunized animals^19^. A different immunization strategy defined by BCG priming followed by a CFP plus CpG boost confers protection against *M. tuberculosis* challenge and induces mild pulmonary inflammation^20^. These clinical and experimental findings show that inflammation, which is closely associated with protective immune responses, is a double-edged sword in tuberculosis pathogenesis and that IFN-γ plays a critical role in this process. Approximately half of the patients who are cured with current tuberculosis drugs suffer tissue damage generated by excessive inflammation^21^. In addition, inflammation may be coopted by anti-inflammatory or regulatory components to counteract the Th1 immune response^22,23^. Therefore, a fine balance between inflammation and regulation of the inflammatory response is imperative for host protection and tissue protection^24^.

CD4^+^Foxp3^+^ T cells inhibit IFN-γ production in patients with active tuberculosis^25,26^. Moreover, regulatory T-cells exacerbate the susceptibility to *M. tuberculosis* infection^27,28^. Pathogen-specific regulatory T cells are capable of delaying the priming of effector CD4^+^ and CD8^+^ T cells in the pulmonary lymph nodes and their subsequent accumulation in the lung^29^. These collective data show that regulatory T cells are detrimental for the control of *M. tuberculosis* infection. Studies on regulatory T cells and tuberculosis have mostly focused on the progression of the infection, but not on the magnitude of pulmonary inflammation.

Because CCR4 induces the recruitment of regulatory T cells to the lung^30–32^, we used CCR4-deficient (CCR4^−/−^) mice as a tool to address the role of CD4^+^Foxp3^+^ T cells in the chronic lung inflammation induced during *M. tuberculosis* infection. CCR4^−/−^ mice exhibited a lower frequency of CD4^+^Foxp3^+^ cells in the early (15 days), initial (30 days), and chronic (70 days) phases of infection than their respective WT counterparts. An increase in lung inflammation and in susceptibility was apparent only at 70 days of infection and was associated with a stronger Th1 immune response. In addition, CCR4^−/−^ mice also exhibited a decrease in the suppressor function of regulatory T cells compared with infected WT mice. Adoptive transfer of Foxp3^+^ cells into infected CCR4^−/−^ mice restored the pulmonary inflammation and bacterial load to levels observed in infected WT mice.

## Results

### CCR4 plays a time-dependent role in *M. tuberculosis* infection

**Fig. 1:**
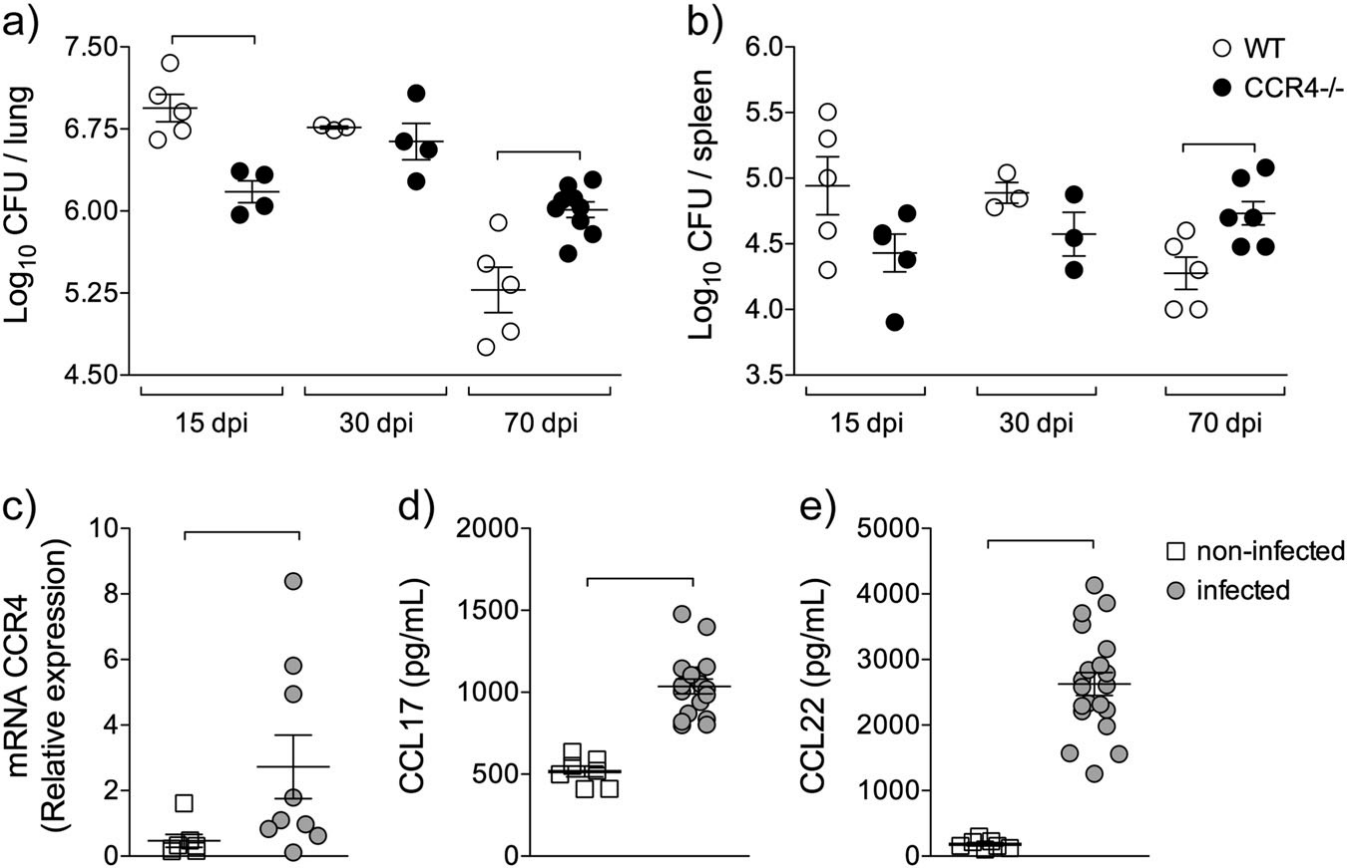
CCR4 affects in a time-dependent way *M. tuberculosis* infection. C57BL/6 (WT) and CCR4^−/−^ mice were infected with 1 × 10^5^ *M. tuberculosis* bacilli by the intra-tracheal route or left non-infected. Bacillus recovery from lungs **a** and spleens **b** of WT (white circles) and CCR4^−/−^ (black circles) mice at 15, 30 and 70 days of infection (dpi). Data are representative of one experiment reproduced three times (*n* = 3–9), expressed as the mean ± SEM. The lungs of non-infected (white squares) and infected (gray circles) mice, at 70 days of infection, were collected. CCR4 gene expression **c**, CCL17 **d** and CCL22 **e** levels were evaluated in lung homogenates. Data are representative of two-independent experiments (*n* = 7–20) expressed as the mean ± SEM. Symbols represent individual animals and bars show the difference (*P* < 0.05) between the groups

To address the contribution of CCR4 during *M. tuberculosis* infection, we first recovered the bacilli from the lungs of infected WT and CCR4^−/−^ mice at 15 (early), 30 (initial) and 70 (chronic) days of infection. At 15 days of infection, CCR4-deficient animals exhibited improved resistance compared with their WT counterparts (Fig. 1a), although there was no difference in the number of bacilli quantified in the spleens of these mice (Fig. 1b). There was also no difference in the colony forming unit (CFU) counts in either the lungs or the spleens at 30 days of infection (Fig. 1a, b). The absence of CCR4 resulted in exacerbated susceptibility to *M. tuberculosis* infection at 70 days, as the CFU counts in the lungs and spleens were significantly higher in CCR4-deficient mice than in their WT counterparts (Fig. 1a, b).

Because CCR4 deficiency increases susceptibility in the chronic phase of infection, we next assessed the gene expression of CCR4 and its ligands CCL17 and CCL22. We found a significant increase in the gene expression of CCR4, CCL17 and CCL22 in the lungs of WT infected mice (TB) compared with non-infected mice (CT) (Fig. 1c–e). Therefore, CCR4 plays a time-dependent role in the lung immune response to *M. tuberculosis* infection.

### CCR4 regulates the magnitude of lung inflammation in the chronic phase of M. tuberculosis infection

**Fig. 2:**
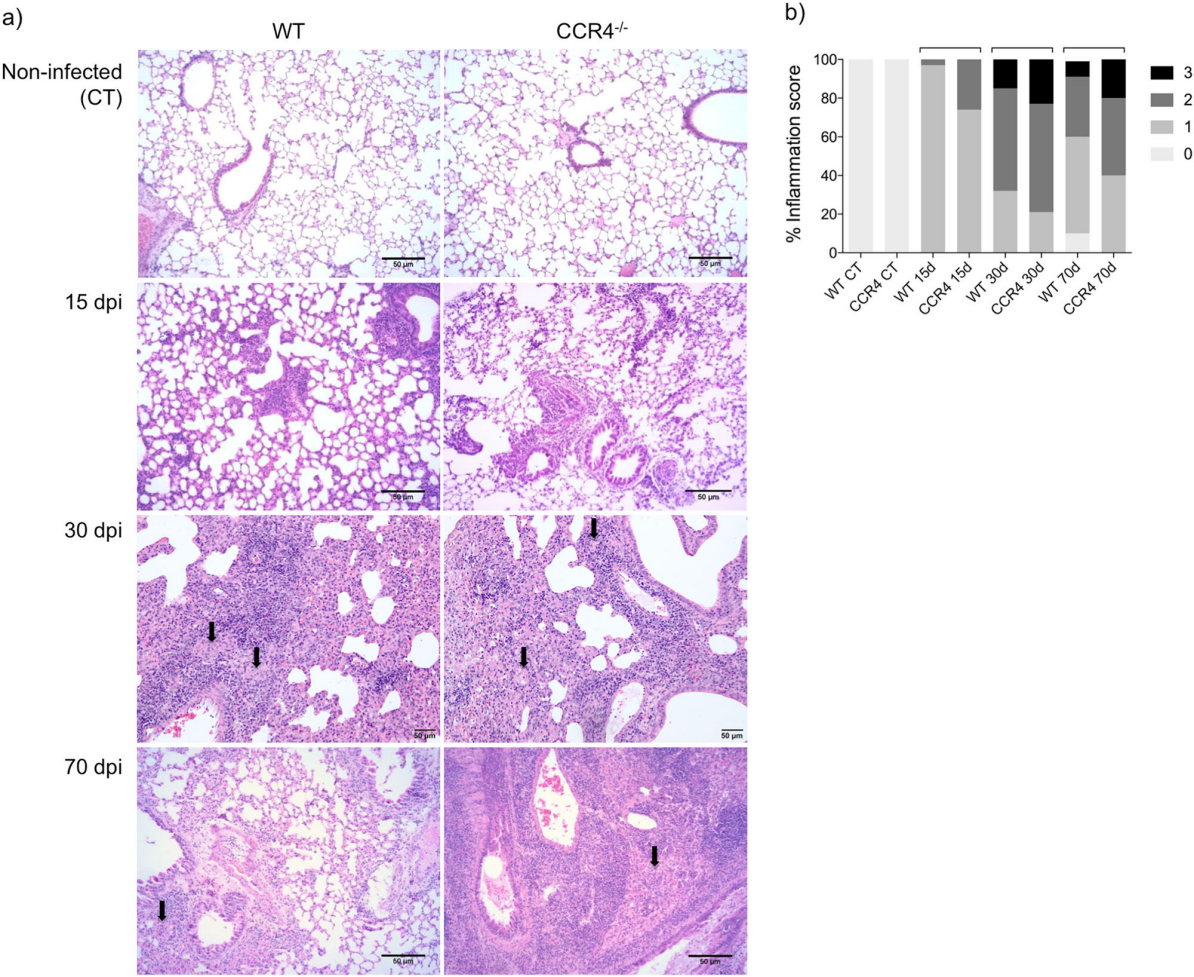
CCR4 regulates the magnitude of the lung inflammation at the chronic phase of *M. tuberculosis* infection. Representative analysis of lung inflammation **a** and inflammation score **b** of WT and CCR4^−/−^ mice were obtained from lung sections stained with hematoxylin and eosin at 15, 30 and 70 days of infection (dpi). Magnification ×200. Data are representative of three independent experiments (*n* = 6–10). Bars show the difference (*P* < 0.05) between the groups. Arrows indicate foamy macrophage clusters

Because, the progression of tuberculosis in immunocompetent subjects is closely associated with pulmonary damage as consequence of the inflammatory response^16^, we next evaluated pulmonary inflammation. As shown in Fig. 2a, at 15 days of infection, lung sections from infected WT and CCR4^−/−^ mice were characterized by focal distribution of predominantly lymphocytes, scattered across bronchioles and vessels. No difference was observed between the two groups.

At 30 days of infection, the magnitude and pattern of inflammation was similar between infected WT and CCR4^−/−^ mice, although the magnitude was higher than that observed in the lungs of mice at 15 days of infection. Small clusters of foamy macrophages were observed (arrows).

At 70 days of infection, larger clusters of foamy macrophages (arrows) and perivascular lymphocyte infiltration were observed in the lungs of WT mice. Although infected CCR4^−/−^ mice exhibited the same pattern of inflammation, the magnitude of the inflammatory response was clearly higher than that found in infected WT mice. Figure 2b shows the lung inflammation scores, which confirm the large areas of inflammation observed in the lungs of CCR4^−/−^ mice at 70 days of infection compared with their WT counterparts.

Together, these findings show that CCR4 contributes to the control of *M. tuberculosis* infection and the regulation of pulmonary inflammation.

### CCR4 increases lung CD4^+^Foxp3^+^ cells during *M. tuberculosis* infection

**Fig. 3:**
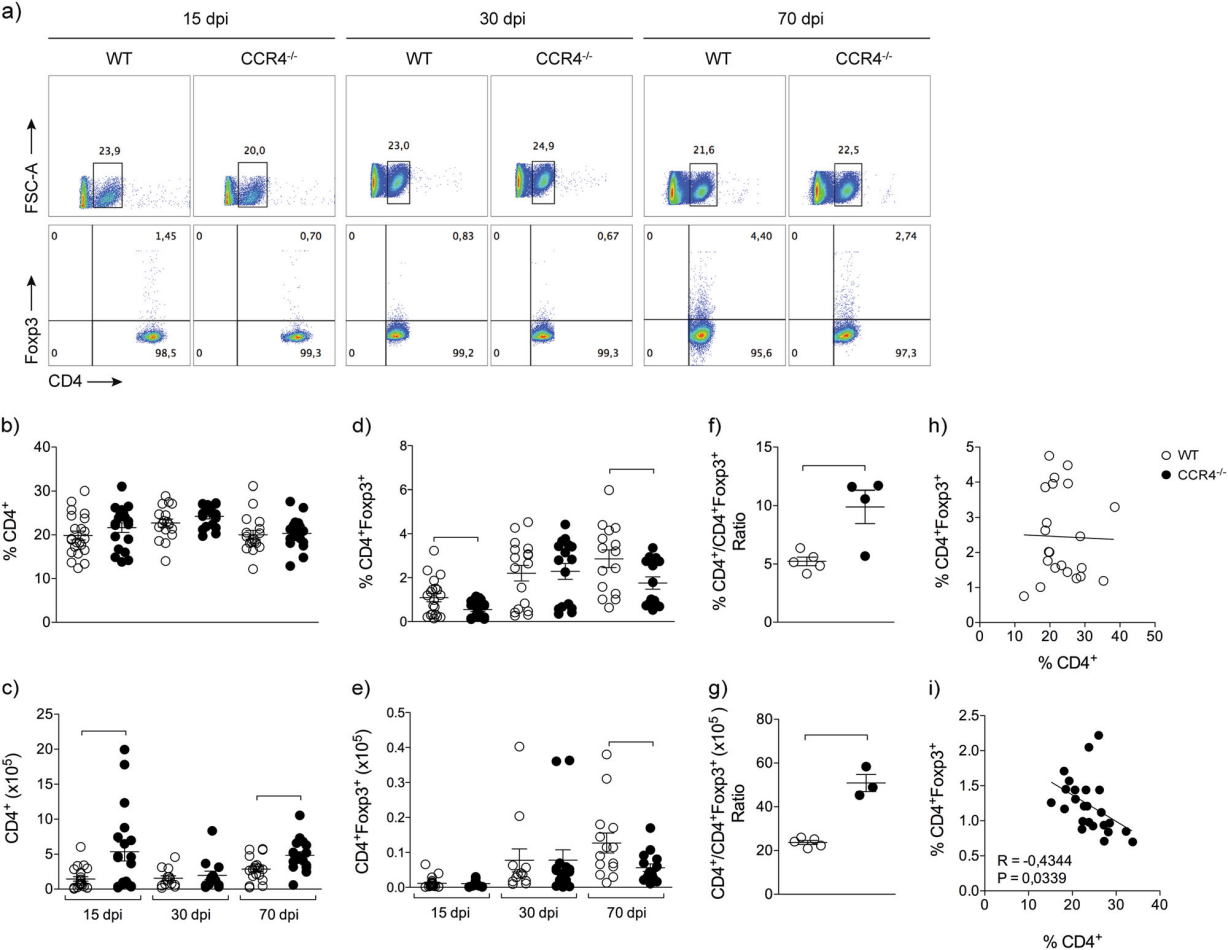
CCR4 increases lung CD4^+^Foxp3^+^ cells during *M. tuberculosis* infection. WT (white circles) and CCR4^−/−^ (black circles) mice were infected with *M. tuberculosis* as described in the Fig. 1. At 15, 30 and 70 days of infection (dpi) the lungs were collected. Representative analysis of CD4^+^ and CD4^+^Foxp3^+^ cells **a**. Frequency and total number of CD4^+^ cells **b**, **c** and CD4^+^Foxp3^+^ cells **d**, **e**. Data are representative of three-independent experiments (*n* = 16–22), expressed as the mean ± SEM. Ratio of frequency **f** and total number **g** of CD4^+^ and CD4^+^Foxp3^+^ cells obtained from lungs of infected WT and CCR4^−/−^ mice. Data are representative of one experiment reproduced four times (*n* = 3–5), expressed as the mean ± SEM. Correlation between the frequency of CD4^+^ and CD4^+^Foxp3^+^ cells from lungs of infected WT and CCR4^−/−^ mice **h**, **i**. Data are representative of four-independent experiments (*n* = 22–25). All data are expressed as the mean ± SEM. Symbols represent individual animals and bars show the difference (*P* < 0.05) between the groups

Considering the increase in the magnitude of lung inflammation in the chronic phase of infection in CCR4^−/−^ mice and the role of CCR4 in the recruitment of regulatory T cells into the lungs, we next quantified these cells. Figure 3a depicts the representative analysis of both CD4^+^ and CD4^+^Foxp3^+^ cells in the lungs of infected mice. The frequency of CD4^+^ cells was similar between infected CCR4^−/−^ and WT groups at 15, 30 and 70 days of infection (Fig. 3b). However, a significant increase in the number of CD4^+^ cells was found in the lungs of infected CCR4^−/−^ animals at 15 and 70 days of infection (Fig. 3c). A decrease in the frequency of CD4^+^Foxp3^+^ cells was found in the lungs of infected CCR4^−/−^ mice at 15 and 70 days of infection compared with their WT counterparts (Fig. 3d), and the number of CD4^+^Foxp3^+^ cells was significantly lower in CCR4^−/−^ mice at 70 days of infection compared with WT mice (Fig. 3e). In addition, we observed a significant increase in the ratio of the frequency or number of CD4^+^/CD4^+^Foxp3^+^ cells obtained from CCR4^−/−^ mice compared with WT mice (Fig. 3f, g). A negative correlation confirmed that as more CD4^+^ cells, lesser CD4^+^Foxp3^+^ cells in the lungs of CCR4^−/−^ mice, but not WT mice, at 70 days of infection (Fig. 3h, i). These results show that in the absence of CCR4, the frequency and number of CD4^+^Foxp3^+^ cells in the lungs are reduced in the chronic phase of infection, followed by an increase in CD4^+^ cells.

### Absence of CCR4 exacerbates lung Th1 inflammation and bacterial load into the lungs

CD4^+^Foxp3^+^ cells are detrimental for the protective immune response against *M. tuberculosis*^25,27–29^, although these cells are important for balancing the inflammatory response^33,34^. Our results above show that the susceptibility to infection, the magnitude of inflammation and the number of CD4^+^Foxp3^+^ cells in the lungs are dependent on CCR4. Because the absence of CCR4 increased the number of CD4^+^ cells in the lungs in the chronic phase of infection, we further evaluated the functional activity of CD4^+^ subsets.

**Fig. 4:**
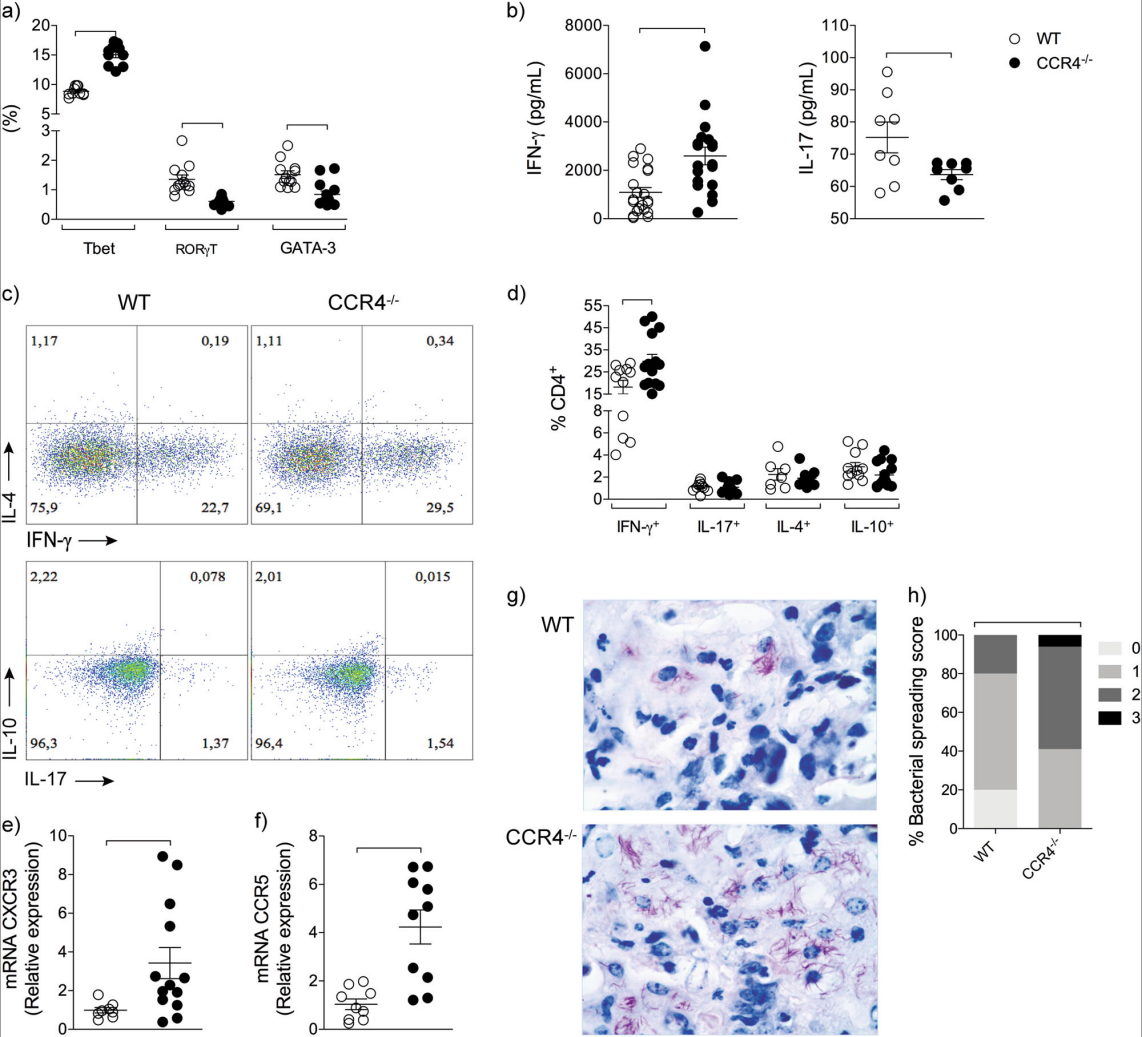
Absence of CCR4 exacerbates lung Th1 inflammation and bacterial spreading into the lungs. WT (white circles) and CCR4^−/−^ (black circles) mice were infected with *M. tuberculosis* as described in the Fig. 1. At 70 days of infection, the lungs were collected. Frequency of lung CD4^+^Tbet^+^, CD4^+^RORγt^+^ and CD4^+^GATA-3^+^ cells **a**. IFN-γ and IL-17 levels in the lung homogenates **b**. Representative analysis of IFN-γ-, IL-4-, IL-17- and IL-10-producing CD4^+^ cells **c**. Frequency of IFN-γ-, IL-4-, IL-17- and IL-10-producing CD4^+^ cells **d**. CXCR3 **e** and CCR5 **f** gene expression in the lung homogenates. Representative Ziehl-Neelsen staining on lung sections at 70 days of infection (magnification, ×400) **g**. Lung bacterial spreading score **h**. Data are representative of three-independent experiments (*n* = 9–22) expressed as the mean ± SEM. Symbols represent individual animals and bars show the difference (*P* < 0.05) between the groups

Among the transcription factors that are master regulators of Th1, Th17 and Th2 responses, we found a significant increase in the frequency of T-bet-expressing CD4^+^ cells and a reduction in the RORγt- or GATA-3-expressing CD4^+^ cells in the lungs of infected CCR4^−/−^ mice compared with WT mice (Fig. 4a). In addition, higher concentrations of IFN-γ and lower levels of IL-17 were detected in the lung homogenates of infected CCR4^−/−^ mice compared with their WT counterparts (Fig. 4b). No differences in the IL-4 and IL-10 levels were found (data not shown). To confirm the increased magnitude of the Th1 immune response in the lungs of infected CCR4^−/−^ mice, we also evaluated the frequency of IFN-γ-producing CD4^+^ cells. Figure 4c shows the representative analysis of cytokine-producing CD4^+^ cells. Infected CCR4^−/−^ mice exhibited a significantly higher frequency of IFN-γ-producing CD4^+^ cells than their WT counterparts and similar frequency of IL-17-, or IL-4-, or IL-10-producing CD4^+^ cells (Fig. 4d). To reinforce the pattern of Th1 inflammation, we evaluated the gene expression of chemokine receptors involved in the recruitment of Th1 cells. The gene expression of both CXCR3 and CCR5 was increased in the lungs of infected CCR4^−/−^ mice (Fig. 4e, f).

In an attempt to associate the broad Th1 inflammation with susceptibility, we found by Ziehl-Neelsen staining more lung bacterial load in CCR4^−/−^ mice (Fig. 4g). This representative result was confirmed by an analysis of bacterial load scores (Fig. 4h).

These collective findings show that CCR4 regulates the frequency of CD4^+^Foxp3^+^ cells in the lungs, which is inversely correlated with the frequency of IFN-γ-producing CD4^+^ cells. Moreover, the exacerbation of lung Th1 inflammation in the absence of CCR4 is associated with increased susceptibility to *M. tuberculosis* infection.

### CCR4 regulates the suppressor function of regulatory T cells during *M. tuberculosis* infection

**Fig. 5:**
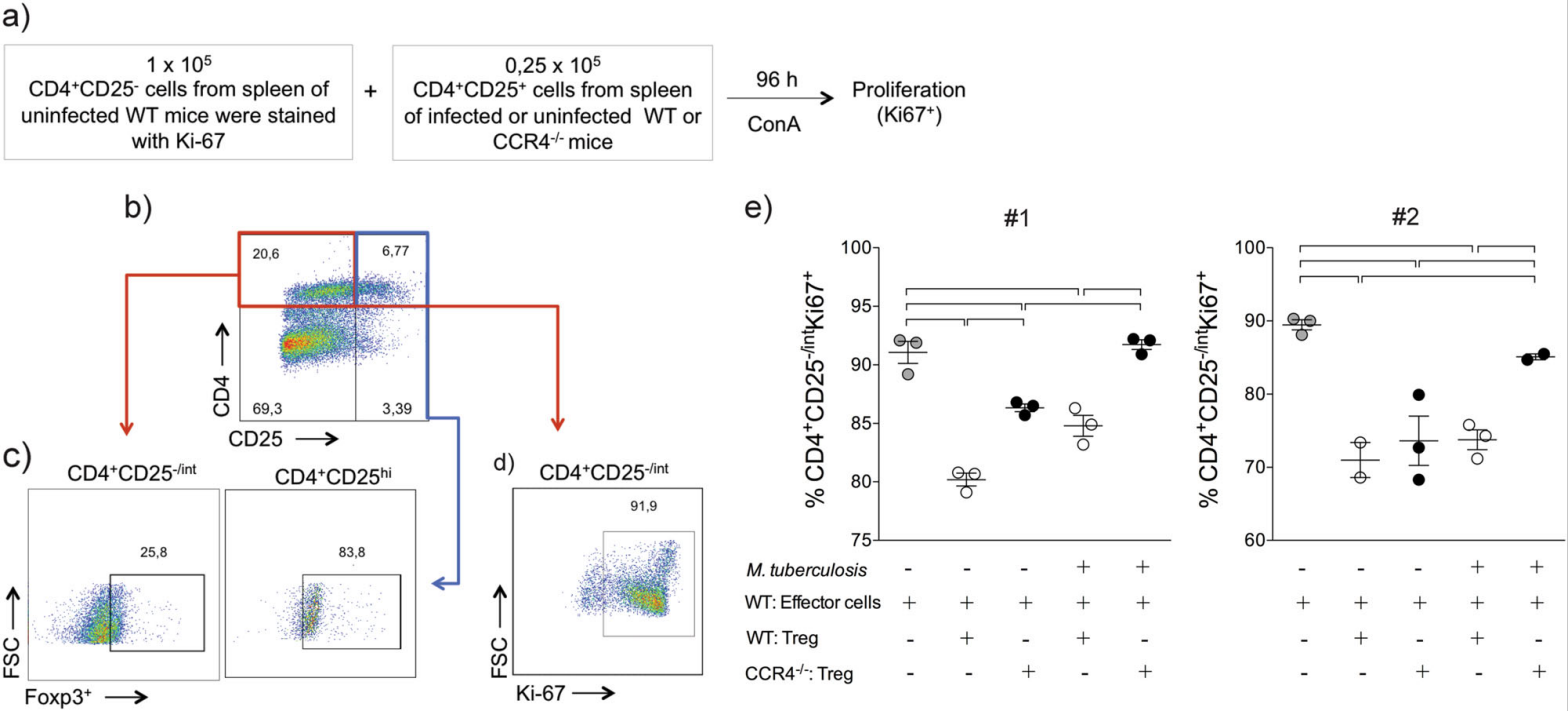
CCR4 regulates the suppressor function of regulatory T cells during *M. tuberculosis* infection. WT (white circles) and CCR4^−/−^ (black circles) mice were infected with *M. tuberculosis* as described in the Fig. 1 or left uninfected. At 70 days of infection, the spleens were collected. CD4^+^CD25^−^ (effector) cells (1 × 10^5^) purified from the spleens of uninfected WT mice were co-cultured with CD4^+^CD25^+^ (regulatory) cells (0.25 × 10^5^) from the spleens of uninfected or infected WT or CCR4^−/−^ mice. Co-cultures were stimulated with ConA and after 96 h, proliferation was assessed **a**. Foxp3 expression on CD4^+^CD25^+^ cells **b**, **c**. Proliferation was assessed by the Ki-67 expression on CD4^+^CD25^−^ cells **d**, **e**. As a positive control, CD4^+^CD25^−^ cells were stimulated with ConA in the absence of CD4^+^CD25^+^ cells (gray circles). Data from two reproduced experiments #1 and #2 (*n* = 3–5) expressed as the mean ± SEM. Symbols represent individual animals and bars show the difference (*P* < 0.05) between the groups

In addition to inducing the recruitment of regulatory T cells into the lungs^32^, CCR4 also plays a role in the suppressor function of Foxp3^+^ cells^31,35^. We next evaluated the suppressor function of regulatory T cells in vitro. CD4^+^CD25^−^(effector) spleen cells obtained from uninfected WT mice were co-cultured with CD4^+^CD25^+^ spleen cells (regulatory T cells) purified from infected WT or CCR4^−/−^ mice or their uninfected counterparts (Fig. 5a). Figure 5b, c shows the gating strategy used to confirm that CD4^+^CD25^−^ cells were Foxp3^−^ and that CD4^+^CD25^+^ cells were mostly Foxp3^+^ regulatory cells. Figure 5d shows the gating strategy used to evaluate the proliferation of CD4^+^CD25^−^ effector cells according to Ki-67 expression. Regulatory T cells purified from infected or non-infected WT mice inhibited the proliferation of effector T cells (white circles). However, regulatory T cells purified from non-infected CCR4^−/−^ mice (black circles) showed a lower suppressor function compared with regulatory T cells obtained from non-infected WT mice. In addition, regulatory T cells purified from infected CCR4^−/−^ mice (black circles) lost their suppressor function, as observed by the proliferation of CD4^+^CD25^−^ cells co-cultured with regulatory T cells from infected CCR4^−/−^ mice, evaluated in two-independent experiments (Fig. 5e). The proliferation of effector cells in the presence of regulatory T cells purified from infected CCR4^−/−^ mice was comparable to the proliferation of effector cells cultured only with polyclonal stimulation, in the absence of regulatory T cells (Fig. 5e).

These results suggest that CCR4 plays a role in the functional activity of regulatory T cells.

### Foxp3-GFP^+^ cell transfer renders CCR4^−/−^ mice more resistant to *M. tuberculosis* infection

**Fig. 6:**
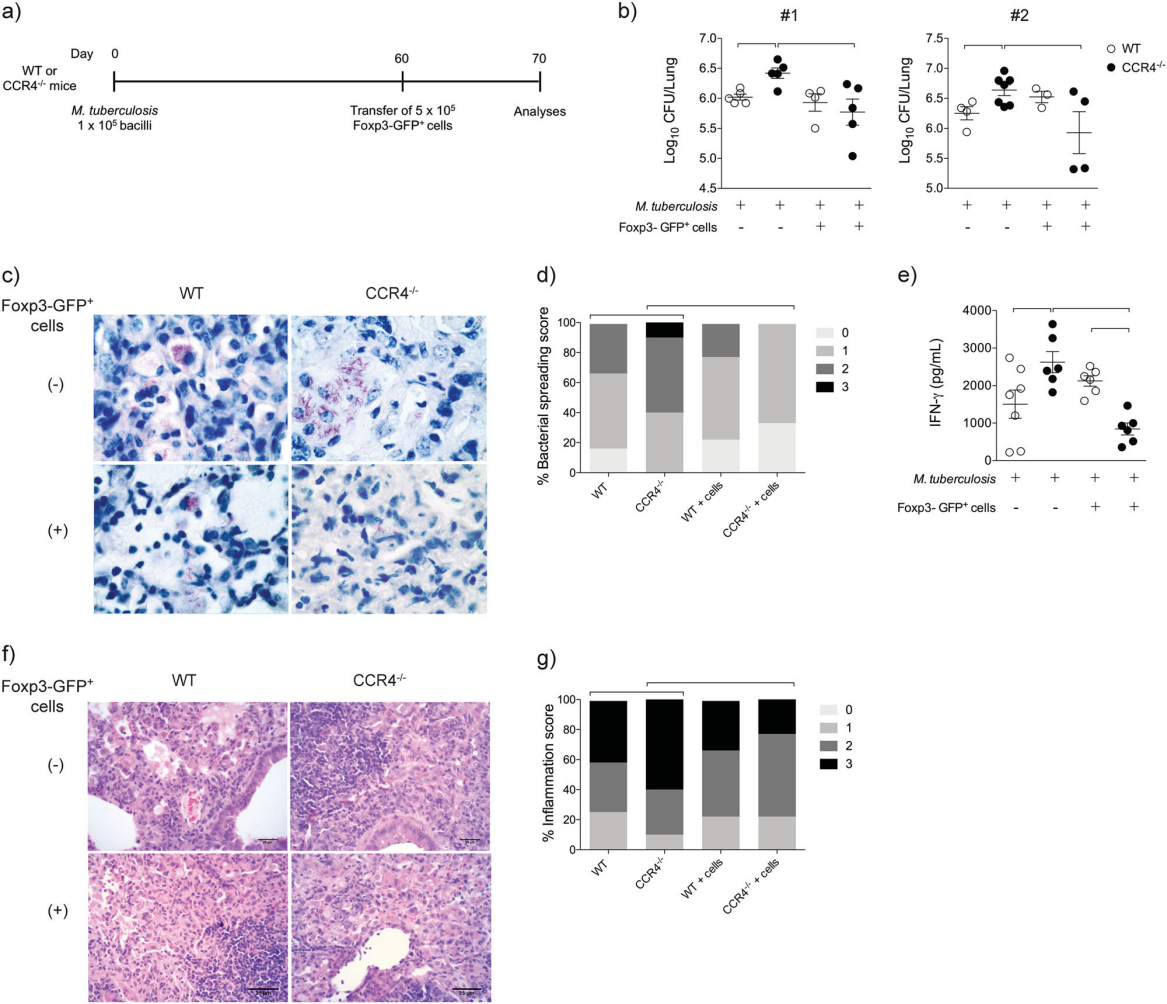
Foxp3-GFP^+^ cell transfer renders CCR4^−/−^ mice more resistant to *M. tuberculosis* infection and ameliorates pulmonary inflammation. WT (white bars) and CCR4^−/−^ (black bars) mice were infected with *M. tuberculosis* as described in the Fig. 1. Spleen Foxp3-GFP^+^ cells (5 × 10^5^) were transferred by the intra-tracheal route to day 60-infected WT or CCR4^−/−^ mice. As an experimental control, infected mice were left without cell transfer. Ten days after Foxp3-GFP^+^ cell transfer, lungs were evaluated **a**. Lung CFU numbers from two reproduced experiments #1 and #2 (*n* = 3–7) expressed as the mean ± SEM **b**. Ziehl-Neelsen staining **c** and lung bacterial spreading score **d**. IFN-γ levels in the lung homogenates **e**. Data are representative of two-independent experiments (*n* = 6–7) expressed as the mean ± SEM. Representative analysis of lung inflammation **f** and inflammation score **g** of WT and CCR4^−/−^ mice were obtained from lung sections stained with hematoxylin and eosin. Magnification ×400. Symbols represent individual animals and bars show the difference (*P* < 0.05) between the groups

We next evaluated the suppressor function of regulatory T cells obtained from infected CCR4^−/−^ mice in vivo. Sorted spleen Foxp3-GFP^+^ cells (5 × 10^5^) from non-infected mice were transferred intratracheally into WT or CCR4^−/−^ mice at 60 days of infection. After 10 days (70 days of infection), the lungs were collected for evaluation (Fig. 6a).

Foxp3-GFP^+^ cell transfer rendered infected CCR4^−/−^ mice, but not infected WT mice, resistant, as observed by the significant reduction in the lung CFU number compared with infected CCR4^−/−^ mice that did not undergo cell transfer (Fig. 6b). No difference in the CFU number was found in the lungs of infected WT mice, regardless of whether Foxp3-GFP^+^ cells were transferred (Fig. 6b). Ziehl-Neelsen staining confirmed the CFU assay findings (Fig. 6c), which were also validated by lung bacillus load scores (Fig. 6d). In addition, Foxp3-GFP cell transfer to infected CCR4^−/−^ mice reduced IFN-γ levels in the lung homogenates (Fig. 6e) and induced mild lung inflammation compared with infected CCR4^−/−^ mice that did not undergo cell transfer (Fig. 6f). The inflammation scores are shown in Fig. 6g.

These results show that CCR4 affects the magnitude of pulmonary inflammation in the chronic phase of *M. tuberculosis* infection and, as a consequence, alters the susceptibility to infection by a mechanism dependent on the balance of CD4^+^Foxp3^+^ regulatory T cells and CD4^+^ Th1 effector cells and on the suppressor function of regulatory T cells.

**Fig. 7:**
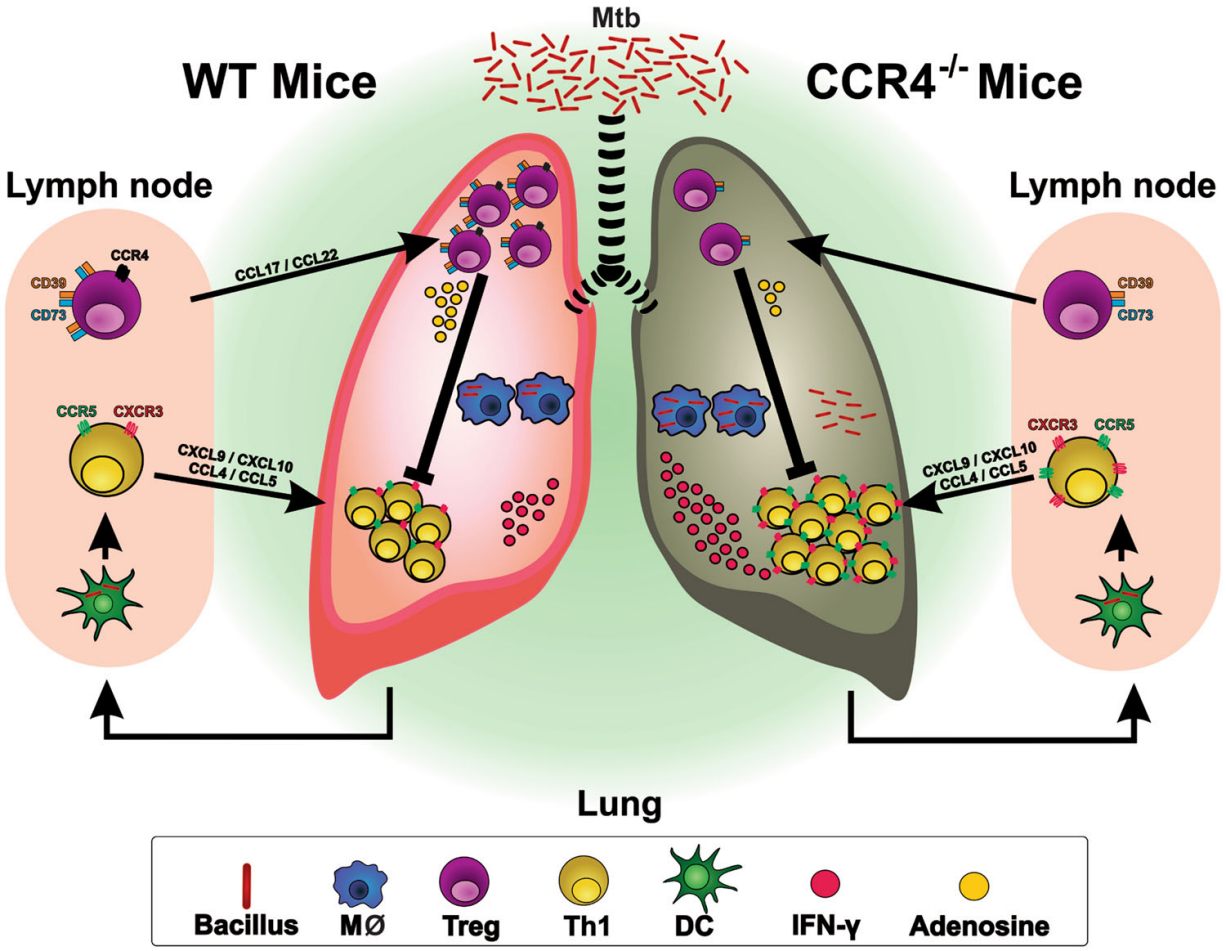
Proposed model of collected findings. CCR4 regulates the magnitude of pulmonary inflammation at the chronic phase of *M. tuberculosis* infection by a mechanism dependent on the balance in the ratio of CD4^+^Foxp3^+^regulatory T cells and CD4^+^ Th1 effector cells, as well as in the suppressor function of regulatory T cells. Consequently, CCR4 deficiency accentuates the susceptibility to infection by a mechanism dependent on exacerbated magnitude of Th1 cell-mediated pulmonary inflammation

Based on our findings, we suggest a model for the role of CD4^+^Foxp3^+^ cells in the balance between inflammatory and anti-inflammatory mechanisms in the chronic phase of *M. tuberculosis* infection. After *M. tuberculosis* infection, dendritic cells migrate to the draining lymph nodes and induce the differentiation of Th1 cells, which are recruited to the lungs via CXCR3 and CCR5. The recruitment of CD4^+^Foxp3^+^ cells via CCR4 maintains the balance between effector and regulatory responses. CCR4 deficiency disrupts the balance between CD4^+^ cells and CD4^+^Foxp3^+^ cells and impairs the suppressor function of regulatory T cells due to the downregulation of CD39 and CD73 (data not shown). Consequently, there is an increased influx of IFN-γ-producing CD4^+^ cells via CXCR3 and CCR5, which reflects the exacerbation of pulmonary inflammation and, indirectly, bacterial spreading and the susceptibility of CCR4^−/−^ mice compared with WT mice. Foxp3^+^ cell transfer restores the balance between effector and regulatory cells and, as a consequence, improves resistance, inducing mild inflammation and lower IFN-γ levels (Fig. 7).

## Discussion

Tuberculosis is responsible for the highest number of infectious-disease-related deaths worldwide^3^. In immunocompetent adults, tuberculosis pathogenesis is closely associated with severe pulmonary inflammation, and IFN-γ is a key molecule in this process, although it is also critical in the protective immune response against mycobacteria^16,36^. CD4^+^Foxp3^+^ regulatory T cells balance the inflammatory response^33,34^. However, CD4^+^Foxp3^+^ cells suppress INF-γ-mediated antimicrobial responses and increase susceptibility to *M. tuberculosis* infection^27–29,37^. Whereas CD4^+^Foxp3^+^ cells regulate inflammation-mediated tissue damage, this aspect has not been addressed during chronic tuberculosis, the hallmark of which is the perpetuation of lung inflammation. In a previous study, we showed that the suppressor function of regulatory T cells during *M. tuberculosis* infection is associated with the host genetic background and suggested that it could represent a susceptibility factor for tuberculosis^28^. Here, we show for the first time that regulatory T cells balance the effector function of Th1 cells in the chronic phase of infection and that this is critical to prevent exacerbation of pulmonary inflammation and, consequently, bacterial growth. Our findings suggest that tuberculosis pathogenesis results from a failure of CD4^+^Foxp3^+^ cell-mediated immune regulation. The absence of CCR4 causes an imbalance in lung CD4^+^ and CD4^+^Foxp3^+^ cells, which exacerbates the Th1-mediated immune response, induces excessive pulmonary inflammation and progression of infection.

We confirmed that regulatory T-cells restrict the protective immune response in the early stage of experimental tuberculosis, as reported previously^29^. The reduction in the accumulation of regulatory T cells at the beginning of the infection, as observed in CCR4^−/−^ mice at 15 days post infection, could minimize the delay in the induction of the adaptive immune response, as has been previously described in tuberculosis^29,38^. Thus, CCR4^−/−^ mice are more resistant than their WT counterparts at 15 days post infection. At 30 days of infection, according to results obtained by our group^39^ animals generate a strong cellular immune response dependent on IFN-γ. However, they are unable to control the infection. Therefore, a reduction in the influx of CD4^+^Foxp3^+^ cells at 30 days of infection does not play an evident role in controlling pathogen replication or the lung inflammatory response. At 70 days of infection, lung CFU number declines in C57BL/6 mice, which are resistant to *M. tuberculosis* infection, but not in CCR4^−/−^ mice. Therefore, an imbalance in the frequency and number of CD4^+^Foxp3^+^ cells, which is associated with the augmentation of Th1 effector cell function, has a deleterious effect on the magnitude of lung inflammation and, consequently, in the infection control.

The identification of key molecules or receptors that can regulate the inflammatory response without affecting antimicrobial mechanisms is crucial for identifying new immunotherapy targets and for the design of host-directed therapies. We recently showed that an increase in CD11c^+^CD206^+^ myeloid cells dependent on the IL-33/ST2 axis and induced by allergen exposure improves the resistance of *M. tuberculosis*-infected mice. We found that M2 macrophages, a phenotype compatible with CD11c^+^CD206^+^ cells, in the presence of IFN-γ exhibit strong mycobactericidal activity and provided insights into the balance of lung inflammation and the maintenance of antimicrobial mechanisms^40^.

In addition to playing a role in the accumulation of CD4^+^Foxp3^+^ cells in the lungs, CCR4 may also affect the function of these cells. Although the transfer of CCR4^+^ regulatory T cells isolated from spleens and lymph nodes protects against the development of colitis, the transfer of CCR4^−^ regulatory T cells fails to prevent the development of disease^35^. Chemokine receptors expressed by regulatory T cells affected their function in vivo by influencing their homing properties. A similar result has been observed in experimental allergic asthma, where the transfer of CCR4^+^ regulatory T cells obtained from spleens and lymph nodes, but not CCR4^−^ regulatory T cells, attenuates eosinophilia, the production of IL-4 and IL-5, and airway inflammation^31^. The exacerbation of allergic inflammation was directly associated with impaired migration of CCR4^−/−^ regulatory T cells to airways and augmented frequency of pulmonary Th2 cells. Therefore, regulatory T cells from CCR4^−/−^ mice are less suppressive, because the absence of CCR4 impairs the migration of these cells to the site of immune response. In addition, the absence of CCR4 impairs that regulatory T cells remain at the site of immune response and, consequently, make an effective contact with dendritic cells^35^. We showed in vitro that regulatory T cells purified from infected CCR4^−/−^ mice lost their suppressor function compared with CD4^+^CD25^+^Foxp3^+^ cells from WT mice. This result shows that CCR4^−^ regulatory T cells induced by infection are less suppressive than CCR4^+^ regulatory T cells obtained from infected animals and natural CCR4^−^ regulatory T cells (obtained from non-infected mice). In vivo, we confirmed by the transfer of Foxp3-GFP^+^ cells to infected CCR4^−/−^ mice that during chronic infection, regulatory T cells contribute to the control of pathogen multiplication, possibly because they balance IFN-γ-mediated inflammation.

The reduction in the frequency, number and function of CD4^+^Foxp3^+^ cells is associated with an increased frequency of IFN-γ-producing CD4^+^ cells and increased IFN-γ levels. Inflammation followed by high levels of IFN-γ contributes to lung injury. Although low-Th1 inflammation and a low capacity to produce IFN-γ are the main causes of tuberculosis pathogenesis in young children and immunodeficient individuals, a high level of lung inflammation and an excessive IFN-γ^+^CD4^+^ cell response are indicators of active disease in immunocompetent adults^15^. As the majority of individuals with tuberculosis are immunocompetent adults, is imperative to understand the regulatory pathways of the inflammatory response during *M. tuberculosis* infection and to identify strategies that simultaneously alleviate inflammation and maximize antimicrobial mechanisms. In summary, an animal model of tuberculosis improves our understanding of the mechanisms that mediate protection against tissue damage and protection against pathogen load. Comorbidities such as type 2 diabetes reinforce the relevance of these findings. Diabetes increases the risk of developing active tuberculosis, in part because type 2 diabetes patients exhibit a level of inflammation, among other alterations, that may increase tuberculosis immunopathology^41–43^.

Our findings suggest that the use of CD4^+^Foxp3^+^ cells or a CCR4 agonist as targets for immunotherapy should take into account the phase of the disease and the definition of parameters for evaluating the level of inflammatory response in the infected individual, considering all forms of *M. tuberculosis* infection.

## Methods

### Animals

Specific-pathogen-free female C57BL/6 (WT) and CCR4-deficient (CCR4^−/−^) mice, 6–8 weeks old, were obtained from the local breeding facility of the Ribeirao Preto Medical School, University of São Paulo (FMRP-USP), Brazil. Foxp3-GFP^+^ transgenic mice were kindly provided by Dr. Bernhard Ryffel from Experimental and Molecular Immunology and Neurogenetics, University of Orleans, France. All animals were maintained under barrier conditions in a level III biosafety laboratory with free access to sterile food and water.

All the experiments were conducted with the approval of the Animal Research Ethics Committee of the Ribeirao Preto Medical School, University of São Paulo (protocol number 135/2012).

### Bacteria and infection

The H37Rv strain of *M. tuberculosis* (American Type Culture Collection 27294, Rockville, MD) was grown in 7H9 Middlebrook Broth (DIFCO Laboratories, Detroit, MI, USA) for 7 days at 37 °C. The culture was collected as previously described^44^. Mice were infected by intra-tracheal administration of 1 × 10^5^ bacilli^45^.

### Colony forming unit assay and processing of lung cells

The lower and middle right lobes of the lungs and the spleen were collected and processed as previously reported^46^. For the colony forming unit (CFU) assay, serial dilutions (100, 1000, 10,000, and 100,000) of digested lungs and spleens were plated on supplemented 7H11 agar medium (Difco, Becton, Dickinson and Company, Le Pont de Chaix, France). The CFUs were counted after 28 days of incubation at 37 °C, and the results were expressed as the log_10_ of CFU/lung and log_10_ of CFU/spleen. After lung and spleen processing, the total cell counts were determined in a Countess™ automated cell counter (Invitrogen Eugene, Oregon, USA).

### Flow cytometry

Lung cells were initially incubated for 40 min at 4 °C with supernatant from 2.4G2 cells (containing anti-FcγRII/III antibodies), followed by incubation with monoclonal antibodies (0.5 µg/1 × 10^6^ cells) for 30 min at 4 °C in total darkness. To characterize lymphocyte populations, the following anti-mouse monoclonal antibodies were used: CD4 (RM4-5), CD25 (PC61), CD39 (Duha59), CD73 (TY/11.8) (BD Biosciences, San Jose, CA, USA) and CCR4 (2G12) (BioLegend, San Diego, CA, USA). After cell surface staining, the cells were stained with antibodies against Foxp3 (MF23), T-bet (O4-46), GATA-3 (L50-823) (BD Biosciences, San Jose, CA, USA), RORγ-t (AFKJS-9) and Ki-67 (SolA15) (eBioscience, San Diego, CA, USA) using a Foxp3/Transcription Factor Staining Buffer Set (eBioscience, San Diego, CA, USA) based on the manufacturer’s instructions. The intracellular detection of cytokines was performed as previously described^46^. Briefly, lung cells were cultured with PMA (100 ng/mL), ionomycin (500 ng/mL) (Sigma-Aldrich, St. Louis, MO) and monensin (BD GolgiStop^TM^, BD Biosciences, San Jose, CA, USA) for 6 h at 37 °C and 5% CO_2_. Next, the cells were collected and fixed with 4% formaldehyde (Labsynth®, Diadema, SP, Brazil) diluted in PBS and incubated for 11 min. Then, 1 mL of PBS was added to the cell suspension, which was incubated for 18 h at 4 °C. For permeabilization, the cell suspension was centrifuged at 350×*g* at 4 °C for 10 min and washed with PBS containing 1% FBS (Gibco®, South American, Brazil) and 0.2% saponin. After washing, the cells were incubated for 20 min at 4 °C with supernatant from 2.4G2 cells, followed by incubation with anti-CD4, anti-IFN-γ (XMG1.2), anti-IL-17 (TC11-18H10) (BioLegend, San Diego, CA, USA), anti-IL-4 (11B11) and anti-IL-10 (JES5^−^16E3) (BD Biosciences, San Jose, CA, USA) antibodies for 30 min at 4 °C in total darkness. Data on cells were acquired by flow cytometry using a BD FACSCanto II instrument (BD Bioscience, Franklin Lakes, NJ, USA). One hundred thousand events per sample were collected, doublet discrimination was performed using Forward Scatter Area (FSC-A) vs. Forward Scatter Area (FSC-H) parameters, and the lymphocytes were gated according to size (FSC-A) and granularity (Side Scatter Area, SSC-A). Analyses of CD4^+^Foxp3^+^ cells were performed within a previous CD4^+^ cell gate. Analyses of IFN-γ-, IL-4-, IL-17-, and IL-10-producing cells were performed within the CD4^+^ cell gate. The data were analyzed by FlowJo 7.6.1 TM Software (Tree Star, Inc., Ashland, Oregon, USA).

### Real time polymerase chain reaction (PCR)

PCR-RT was performed as previously described^46^. The samples were analyzed using the Ct (Threshold Cycle) method. Gene expression was calculated as 2^−(ΔΔCt)^, in which ΔΔCt = ΔCt (sample)–ΔCt (calibrator) and ΔCt (sample) = Ct (target gene) – Ct (normalizer = β-Actin). The following primer sequences were used: β-actin (sense: CCCTAGGCACCAGGGTGTGA, anti-sense: GCCATGTTCAATGGGGTACTTC), CCR4 (sense: CGATTCCAAAGATGAATGCCA, anti-sense: TCCCCAAATGCCTTGATACC), CXCR3 (sense: AACGTCAAGTGCTAGATGCCT, anti-sense: TCTCGTTTTCCCCATAATCG) and CCR5 (sense: TGCACAAAGAGACTTGAGGCA, anti-sense: AGTGGTTCTTCCCTGTTGGCA). The primers for CCR4, CCL17, CCL22, CXCR3, and CCR5 were kindly provided by Dr. João Santana da Silva (FMRP-USP).

### Cytokine production

Concentrations of IFN-γ and IL-17 were determined by ELISA using the immunoassay kit BD *OptEIA*^*TM*^ *Set* (BD Biosciences, San Diego, CA, USA) and *DuoSet®* ELISA *Development System* (R&D Systems, Minneapolis, MN, USA), respectively, according to the manufacturer’s instructions. The limits of sensitivity for the assays were 31.3 pg/mL for IFN-γ and 15.6 pg/mL for IL-17.

### Isolation of spleen CD4^+^CD25^−^ and CD4^+^CD25^+^ cells

CD4^+^CD25^+^ and CD4^+^CD25^−^ cells were purified from the total spleen cell population using a MACS CD4^+^CD25^+^ Regulatory T-cell Isolation Kit (Miltenyi Biotec, Bergisch Gladbach, Germany) according to the manufacturer’s instructions.

### Proliferation analysis

After purification of spleen cells, 1 × 10^5^ CD4^+^CD25^−^ cells and 0.25 × 10^5^ CD4^+^CD25^+^ cells (ratio of 4:1) were co-cultured in a 96-well plate (BD, Franklin Lakes, NJ, USA). The co-culture was stimulated with Concanavalin A (ConA; 40 µg/mL) at 37 °C and 5% CO_2_ for 96 h. As a positive control, only CD4^+^CD25^−^ cells were stimulated, and as a negative control CD4^+^CD25^−^ cells were left non-stimulated. The proliferation was evaluated by Ki-67 expression.

### Transfer of Foxp3-GFP^+^ cells

Foxp3-GFP^+^ regulatory T cells were isolated from the spleens of non-infected mice by sorting according to GFP fluorescence using a FACSAria™ Cell Sorter (BD, San Jose, CA, USA). Next, 5 × 10^5^ Foxp3-GFP^+^ regulatory T cells (50 μL) were adoptively transferred intratracheally into mice at 60 days post infection, and 10 days after cell transfer, the number of CFUs was evaluated.

### Histopathology

The right upper lobes of the lungs were fixed in 3.7% formaldehyde, embedded in paraffin blocks and then sectioned for light microscopy. For the histopathological analysis, sections of 5 μm were stained with hematoxylin and eosin (HE) to characterize the cellular infiltrate. The scores were determined as follows: grade 0—absence or presence of rare inflammatory cells; grade 1—a perivascular or peri-bronchial collection of inflammatory cells, predominantly lymphocytes, sometimes forming sparse lymphoid aggregates; grade 2—a perivascular or peri-bronchial collection of inflammatory cells, predominantly lymphocytes and xanthomatous macrophages, forming frequent lymphoid aggregates, with some alveolar spaces preserved; and grade 3—a perivascular or peri-bronchial collection of inflammatory cells, predominantly lymphocytes and xanthomatous macrophages, forming frequent lymphoid aggregates and mostly coalescing, with only a few alveolar spaces preserved.

### Ziehl-Neelsen staining

Lung tissue sections were deparaffinized, washed with decreasing concentrations of alcohol (from 96 to 70% ethanol), and stained with carbol-fuchsin (Merck Millipore, Darmstadt, HE, Germany) for 30 min, followed by incubation with 1% acid alcohol until the staining was completely dissolved and removed. Counterstaining was performed with a methylene blue solution for 2–3 min, followed by washing under running water and decreasing concentrations of alcohol. The slides were mounted with Entellan New ® mounting medium (Merck Millipore, Darmstadt, HE, Germany). The scores were determined as follows: grade 0—absence of bacilli; grade 1—presence of rare bacilli; grade 2—occasional agglomerations of bacilli; grade 3—frequent agglomerations of bacilli.

### Statistical analysis

The data were analyzed with PRISM 5.0 software (GraphPad Software, Inc., San Diego CA, USA). First, the data normality was evaluated by the Kolmogorov-Smirnov test. Next, the statistical significance between two groups was estimated using a two-tailed *t*-test for parametric data and a Mann–Whitney *U*-test for non-parametric data. The data from experiments with three or more groups were analyzed using one-way ANOVA with the Tukey test for parametric data and the Kruskal–Wallis test for non-parametric data. The data are expressed as the mean ± standard error of mean. Values of *P* < 0.05 were considered significant. Correlations were determined by Pearson’s correlation coefficient.
